# Early versus late silicone oil tamponade removal after rhegmatogenous retinal detachment: a retrospective real world comparative study

**DOI:** 10.1186/s40942-025-00743-9

**Published:** 2025-10-13

**Authors:** Nefeli Eleni Kounatidou, Luca Mautone, Vasyl Druchkiv, Martin Stephan Spitzer, Christos Skevas

**Affiliations:** Department of Ophthalmology, University Medical Center Hamburg-Eppendorf, Martinistr. 52, 20246 Hamburg, Germany

**Keywords:** Silicone oil removal, Rhegmatogenous retinal detachment, Endotamponade, Retinal redetachment

## Abstract

**Background:**

To evaluate postoperative outcomes, including redetachment rates, in eyes with rhegmatogenous retinal detachment (RRD) following early or late removal of silicone oil (ROSO).

**Methods:**

This retrospective single-center study included 58 pseudophakic eyes that underwent 23-gauge pars plana vitrectomy (PPV) with silicone oil endotamponade (SOE). Patients were divided into two groups: Group I (ROSO ≤ 12 weeks) and Group II (ROSO > 12 weeks) with SOE duration of 2.3 +/- 0.7 months and 5.2 +/- 1.9 months, respectively. Clinical outcomes, including redetachment rates, best corrected visual acuity (BCVA), cystoid macular edema (CME), epiretinal membrane (ERM) formation, and intraocular pressure (IOP) changes, were analyzed. Kaplan-Meier estimation and Log-Rank tests were used to assess redetachment.

**Results:**

Redetachment occurred in a total of 13 eyes (22% of patients) (Group I: 7 (21%), Group II: 6 (23%); *p* = 1.00). The cumulative incidence of redetachment was comparable between groups (Log-Rank test: *p* = 0.88). Final BCVA at the last follow-up showed no significant difference between Group I (0.8 ± 0.5 logMAR) and Group II (0.8 ± 0.7 logMAR; *p* = 0.36). Postoperative incidences of CME (Group I: 13 (41%), Group II: 9 (35%) *p* = 0.78), ERM (Group I: 13 (41%), Group II: 7 (27%) *p* = 0.40), and elevated IOP (Group I: 7 (22%), Group II: 7 (27%) *p* = 0.76) were also comparable between groups.

**Conclusions:**

Early ROSO (≤ 3 months) does not appear to increase the risk of retinal redetachment or adversely affect visual and anatomical outcomes. These findings support the feasibility of earlier ROSO in selected cases, potentially reducing the risk of silicone oil-related complications.

**Supplementary Information:**

The online version contains supplementary material available at 10.1186/s40942-025-00743-9.

## Introduction

Since its introduction into vitreoretinal surgery by Cibis, silicone oil (SO) has become a standard technique in the management of retinal detachment (RD) [1]. The choice between silicone oil (SO) and other intraocular tamponades, such as SF₆ and C₃F₈ gas, varies considerably depending on the surgeon’s preference and patient-specific factors. Silicone oil endotamponade (SOE) is generally preferred in complicated RDs, such as those associated with established proliferative vitreoretinopathy (PVR), or in cases with a higher incidence of PVR development, including giant retinal tears, signs of uveitis, or preoperative choroidal detachment [2, 3].

Once anatomical success is achieved, the removal of silicone oil (SO) is generally recommended, as prolonged silicone oil endotamponade (SOE) has been linked to ocular complications. These include glaucoma – primarily due to SO emulsification – corneal endothelial decompensation [4, 5], and silicone oil-related visual loss (SORVL) [6]. Notably, some studies have demonstrated a correlation between the duration of SOE and the incidence of ocular complications [6, 7].

The optimal timing for removal of silicone oil (ROSO) remains unclear, with most surgeons recommending removal within 3 to 6 months. Some series suggest a higher risk of retinal redetachment when the tamponade is maintained for less than 3 months, whereas others have found no association between tamponade duration and redetachment rates [8–11]. Moreover, other studies have demonstrated favorable anatomical and functional outcomes even with shorter durations of SOE [11–14]. Interestingly, early ROSO – before the commonly preferred duration of 3-months-period – is increasingly recommended in selected cases. This is due to the decreased incidence of complications, such as SORVL, with short-term endotamponade durations of 2 months or less [6]. In fact, some reports indicate that the first signs of silicone oil (SO) emulsification — the primary factor associated with SO-related intraocular pressure (IOP) elevation — can already appear within the first 3 months after implantation [15].

The aim of this retrospective study is to compare postoperative outcomes, including redetachment rates, in eyes with rhegmatogenous retinal detachment following ROSO before versus after the generally preferred 3-month endotamponade period.

## Materials and methods

This retrospective single-center, comparative study included eyes of pseudophakic patients with rhegmatogenous retinal detachment (RRD) treated with 23G pars plana vitrectomy (PPV) and SOE at the Department of Ophthalmology of the University Medical Center Hamburg-Eppendorf, Hamburg, Germany. The electronic medical records (ifa Systems AG, Germany) from January 2016 to December 2023 were reviewed. The study complied with the Declaration of Helsinki Statement for Trials and was evaluated by the Institutional Review Board of the Hamburg Medical Chamber (*Ethik-Kommission der Ärztekammer Hamburg*); ethical approval was waived and granted due to the retrospective nature of the study.

For patient identification, the electronic database search function was used with the following terms: “rhegmatogenous detachment”, “retinal detachment”, “retinal tear”, “macula on retinal detachment”, “pars plana vitrectomy”, “vitrectomy”, “silicone oil endotamponade”, “silicone oil removal”, “silicone oil”, “surgical management”, “endotamponade”, “pseudophakic”.

**Fig. 1:**
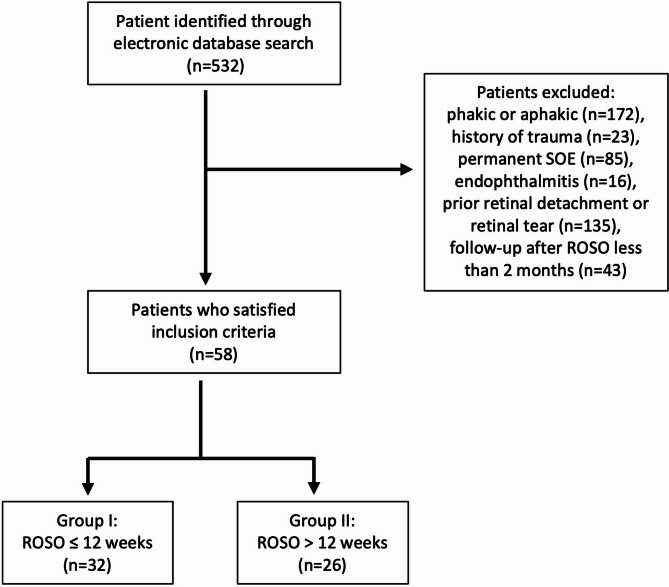
Cohort flow diagram of the study sample. SOE: silicone oil endotamponade, ROSO: risk of silicone oil removal

Patients were excluded if they had a history of perforating trauma, endophthalmitis, prior retinal detachment or tears, syndromes predisposing to a higher RD risk (such as Marfan and Stickler Syndrome), proliferative diabetic retinopathy, prior intraocular surgeries other than extracapsular cataract surgery, or visual acuity-limiting pathology. Only patients with at least two months of follow-up after ROSO were included (Fig. 1). Two independent researchers (C.S. and N.E.K.) screened the records, with any disagreements resolved by discussion or third-party adjudication.

The screening procedure resulted in the inclusion of 58 eyes of 53 patients. The patients were divided into two groups based on the timing of ROSO: Group I included those who underwent ROSO before or during the 12th postoperative week, and Group II included those who underwent ROSO after the 12th postoperative week. The 12-week (or 3-month) cutoff was chosen because it is generally considered the minimum acceptable duration for SOE by most surgeons, as shorter durations have been associated with higher redetachment rates [8, 16]. The primary outcome of this study was to evaluate whether a SOE duration ≤ 3 months could achieve comparable or better anatomical and functional results in comparison to SOE >3 months [6, 15].

For all included eyes, data were extracted into a Microsoft Excel 365 worksheet. Extracted variables included patient demographics, best corrected visual acuity (BCVA) at presentation, before ROSO, and at final follow-up, time between primary ppV and ROSO, intraocular pressure (IOP), use of IOP- lowering medications before and after ROSO, and the incidence and number of RD after ROSO. Elevated IOP was defined as an IOP greater than 21 mmHg in 3 consecutive measurements either before or after ROSO. Eyes with IOP controlled (under 21 mmHg) with topical IOP-lowering medications were also classified as having elevated IOP, consistent with previous studies [17, 18].

In addition to medical records, imaging data from swept-source optical coherence tomography (OCT, DRI OCT Triton, Topcon, Tokyo, Japan) and ultra-widefield retinal images (Optos PLC, Dunfermline, United Kingdom) were analyzed. Data collected included macular status at primary RRD or consecutive RDs after ROSO (macula-on vs. macula-off RD), the presence of postoperative cystoid macular edema (CME) before and after ROSO, the presence of epiretinal membrane (ERM) before and after ROSO, number and location of retinal breaks at initial RRD, bullous morphology of the primary RRD, as previously defined in literature [19], and PVR grading according to the Updated Retina Society Classification (1991) [20]. Surgical protocols for the initial pars plana vitrectomy (PPV) and for ROSO were reviewed, documenting the type of endotamponade used and whether ERM, and/or peripheral membrane peeling, retinectomy or retinotomy were performed.

All included pseudophakic patients in the study underwent 23-gauge ppV with a wide-angle viewing system followed by SO implantation by two experienced surgeons (C.S., M.S.). Prophylactic 360-degree laser retinopexy was applied in all cases. Additional procedures, such as ERM peeling, peeling of peripheral membranes, retinectomy, and/or retinotomy, were performed as needed. The D.O.R.C. silicone oil (Sil-2000-S or Sil-5000-S; D.O.R.C. International, Zuidland, The Netherlands) was used, with the viscosity (2000 or 5000 cSt) selected according to the surgeon’s preference.

ROSO was performed at least one month after the initial PPV. The ROSO procedure involved included 23-gauge ppV with at least three cycles of air-fluid exchange with aspiration of silicone oil using a thin-walled 18- or 19-gauge needle. In selected cases, additional surgical interventions, such as laser photocoagulation, ERM peeling, and peeling of peripheral membranes were performed in some cases. The use of C3F8 gas instead of air during oil removal was performed in certain eyes.

The primary outcome of the study was the comparison of redetachment rates between groups. Secondary outcomes included the incidence of postoperative complications — such as epiretinal membrane formation, CME, and elevated intraocular pressure (IOP)—as well as visual outcomes, in the early (≤ 3 months) and late (> 3 months) ROSO groups. Statistical analyses were performed using R Core Team 2021 (R: Foundation for Statistical Analysis, Vienna, Austria). Fischer’s exact and Mann-Whitney tests were used for demographic comparisons. data, and a Kaplan-Meier estimation and Log-Rank test were employed to compare the incidence of RD after ROSO, as well as preoperative and postoperative incidences of CME, ERM development, and ocular hypertension between groups. Analysis of Variance ANOVA was applied to calculate the effect of time on visual acuity development between groups. In order to minimize baseline imbalances and reduce confounding by indication a matching strategy was further employed. Candidate variables for matching were prespecified based on clinical relevance and included baseline BCVA, macula status at index repair, PVR grade, number of retinal breaks, silicone oil viscosity, tamponade at ROSO, surgeon, and additional maneuvers at ROSO (ILM/ERM peeling, retinectomy). Using the cardinality matching method implemented in the *MatchIt* package in R, the largest subset of eyes that could be balanced across these covariates was selected. The significance level was set at *p* < 0.05.

## Results

### Demographics and baseline characteristics at presentation

A total of 358 patients were identified and screened for eligibility. Ultimately 58 eyes from 53 patients (average age 63.5±14.1, range 15–87) years, *n* = 23 (43.3%) females and *n* = 30 (56.7%) males were included. Group I comprised 33 eyes, and Group II comprised 26 eyes. The median time to the removal of silicone oil (ROSO) was 2.3 ± 0.7 months (range 1.0–3.0) in Group I and 5.2 ± 1.9 months (range 4.0–10.0) in Group II. Subjects were followed for a mean duration of 17.7 ± 14.5 months (range 2.0–59.0) after ROSO.

As shown in Table 1, the groups did not significantly differ in demographic characteristics and morphological findings at presentation. Regarding the initial RRD characteristics, there were no statistically significant differences between the groups in macular involvement (*p* = 1.00), presence of retinal breaks (*p* = 0.07), bullous RRD (*p* = 1.00), extent of retinal quadrant involvement (median 2.0 quadrants; range 2.0–3.0 quadrants; *p* = 0.22), presence of vitreous hemorrhage (*p* = 0.25), or PVR grades (*p* = 0.91). However, the baseline best-corrected visual acuity (BCVA) was better in Group I eyes (1.1 ± 0.8 logMAR; range 0.1–2.4) compared to Group II eyes (1.7 ± 0.8 logMAR; range 0.2–2.7; *p* = 0.02).

**Table 1:** Demographic characteristics and baseline characteristics of the first retinal detachment

	Group I [min, 3 Months] (*N* = 32) *N* (%)	Group II (3 Months, max] (*N* = 26) *N* (%)	Total (*N* = 58) *N* (%)	Standardized Mean Difference	*P* value
**Sex (Eyes)**					0.426^a^
Female	15 (46.9%)	9 (34.6%)	24 (41.4%)	-0.26	
Male	17 (63.3%)	17 (61.3%)	34 (62.3%)	0.26	
**Age (years)**					0.340^b^
Range	38.46–85.49	15.29–87.03	15.29–87.03		
Mean (SD)	65.55 (12.11)	60.97 (16.17)	63.50 (14.13)	-0.28	
Median (Q1, Q3)	66.18 (59.10, 74.23)	62.56 (56.65, 71.15)	63.78 (76.92, 72.15)		
**BCVA at presentation** **(LogMar)**					0.028^b^
Range	0.10–2.40	0.20–2.70	0.10–2.70		
Mean (SD)	1.15 (0.85)	1.70 (0.86)	1.39 (0.89)	0.64	
Median (Q1, Q3)	1.00 (0.40, 2.10)	2.10 (1.00, 2.40)	1.45 (0.60, 2.30)		
**Macula Involvement** **(Primary Retinal Detachment)**					1.000^a^
Macula-Off	22 (68.8%)	17 (68.0%)	39 (68.4%)	-0.02	
Macula-On	10 (31.2%)	8 (32.0%)	18 (31.6%)	0.02	
**Bullous RRD**					1.000^a^
Yes	16 (50.0%)	13 (50.0%)	29 (50.0%)	0.00	
No	16 (50.0%)	13 (50.0%)	29 (50.0%)	0.00	
**PVR Grade**					0.917^a^
A	16 (50.0%)	15 (57.7%)	31 (53.4%)	0.16	
B	8 (25.0%)	6 (23.1%)	14 (24.2%)	-0.05	
C	8 (25.0%)	5 (19.2%)	13 (22.4%)	-0.15	
**Retinal Quadrant Involved (n)**					0.227^b^
Range	1.00–4.00	1.00–4.00	1.00–4.00		
Mean (SD)	2.25 (0.95)	2.58 (1.06)	2.40 (1.01)	0.31	
Median (Q1, Q3)	2.00 (2.00, 3.00)	2.00 (2.00, 3.75)	2.00 (2.00, 3.00)		
**Retinal Breaks (n)**					0.072^b^
Range	0.00–9.00	0.00–5.00	0.00–9.00		
Mean (SD)	1.75 (2.02)	0.96 (1.40)	1.40 (1.80)	-0.56	
Median (Q1, Q3)	1.00 (0.00, 2.00)	0.00 (0.00,1.75)	1.00 (0.00, 2.00)		
**Months to ROSO**					< 0.001^b^
Range	1.0–3.0	4.0–11.0	1.0–11.0		
Mean (SD)	2.3 (0.7)	5.2 (1.9)	3.7 (2.0)	1.64	
Median (Q1, Q3)	3.0 (2.0, 3.0)	4.5 (4.0, 5.0)	3.5 (3.0, 4.2)		
**Presence of vitreous hemorrhage**					0.253^a^
Yes	3 (9.7%)	0 (0.0%)	3 (5.6%)		
No	28 (90.3%)	23 (100.0%)	57 (94.4%)		
**Follow-up (months)**					0.913^b^
Range	2.0–59.0	3.0–57.0	2.0–59.0		
Mean (SD)	17.7 (14.4)	17.8 (14.8)	17.7 (14.5)		
Median (Q1, Q3)	13.0 (8.2, 25.8)	11.0 (6.0, 29.2)	12.5 (6.0, 27.8)		

BCVA: best corrected visual acuity; RD: retinal detachment; PVR: proliferative vitreoretinopathy; RD: retinal detachment; ROSO: removal of silicone oil; a: Fisher’s exact test; b: Mann-Whitney test. Standarized Mean Difference is calculated using standard deviation of the (3 Months, max] group

### Characteristics at primary ppV and ROSO surgery

The primary treatment for RRD included 23-gauge ppV with 360^o^ laser coagulation followed by SOE in all cases. Two surgeons performed all procedures, with no significant difference in the distribution of cases between the groups (*p* = 0.24). Silicone oil (SO) with a viscosity of 2.000 centistokes was used in 24 cases in Group I and 13 cases in Group II, while 5.000 centistoke SO was used in 8 cases in Group I and 13 cases in Group II with usage rates being comparable between the two groups (*p* = 0.06). In our cohort, no intraoperative dyes such as triamcinolone acetonide, tryptan blue or internal limiting membrane staining dyes were used. In certain cases, pefluorocarbon liquid (PCFL) was used at comparable rates between the two groups. Additional procedures, including epiretinal membrane (ERM) peeling, peripheral membranes peeling, retinotomy, and/or retinectomy, were performed in 40 cases without significant intergroup rate differences (Table 2).

**Table 2:** Characteristics of primary pars plana vitrectomy surgery

	Group I [min, 3 Months] *N* (= 32) *N* (%)	Group II [3 Months, max] *N* (= 26) *N* (%)	Total (*N* = 58) *N* (%)	*P* value
**Type of SOE**				0.059^a^
SO (2000 centistokes)	24 (76.6%)	13 (70.0%)	37 (63.8%)	
SO (5000 centistokes)	8 (23.4%)	13 (30.0%)	21 (36.2%)	
**Retinotomy (Primary Surgery)**				0.448^a^
Performed	0 (0.0%)	1 (3.8%)	1 (1.7%)	
Not Performed	32 (100.0%)	25 (96.2%)	57 (98.3%)	
**Retinectomy (Primary Surgery)**				1.000^a^
Performed	0 (0.0%)	0 (0.0%)	0 (0.0%)	
Not Performed	32 (100.0%)	26 (100.0%)	58 (100.0%)	
**ERM Peeling (Primary Surgery)**				1.000^a^
Performed	1 (3.2%)	1 (3.8%)	2 (3.4%)	
Not Performed	32 (96.8%)	25 (96.2%)	57 (96.6%)	
**Peeling of Peripheral Membranes (Primary Surgery)**				0.788^a^
Performed	21 (65.5%)	16 (61.5%)	37 (63.8%)	
Not Performed	11 (34.5%)	10 (38.5%)	21 (36.2%)	
**Pefluorocarbon liquid (PCFL)**				0.743^a^
Used	6 (20.0%)	6 (26.1%)	12 (22.6%)	
Not used	24 (80.0%)	17 (73.9%)	41 (77.4%)	
**Surgeon**				0.245^a^
C.S.	29 (90.6%)	26 (100.0%)	55 (94.8%)	
M.S.	3 (9.4%)	0 (0.0%)	3 (5.6%)	

SOE: Silicone Oil Endotamponade; ERM: epiretinal membrane; a: Fisher’s exact test

ROSO surgery was performed after a minimum SOE duration of at least one-month following confirmation of anatomical success through ophthalmologic examination, OCT, and ultra-widefield retinal imaging. Table 3 summarizes the characteristics before ROSO surgery. All ROSO surgeries were performed by one surgeon (C.S.). Pre- ROSO BCVA was slightly better in Group I (0.7 ± 0.48 logMAR; range 0.20–2.4) than in Group II (0.9 ± 0.5 logMAR; range 0.00–2.70; *p* = 0.05). The incidence of cystoid macular edema (CME), ERM, and ocular hypertension before ROSO did not significantly differ between groups (*p* = 0.16, 0.41, and 1.00, respectively).

**Table 3:** Patient characteristics before ROSO

	Group I [min, 3 Months] *N* (= 32) *N* (%)	Group II [3 Months, max] *N* (= 26) *N* (%)	Total (*N* = 58) *N* (%)	*P* value
**BCVA before ROSO (LogMar)**				0.052^b^
Range	0.20–2.40	0.00–2.70	0.00–2.70	
Mean (SD)	0.72 (0.48)	0.97 (0.59)	0.84 (0.54)	
Median (Q1, Q3)	0.60 (0.40, 1.00)	1.00 (0.50, 1.22)	0.70 (0.50, 1.00)	
**CME before SOR**				0.164^a^
Yes	14 (43.8%)	6 (23.1%)	20 (34.5%)	
No	18 (56.2%)	20 (76.9%)	38 (65.5%)	
**ERM before SOR**				0.417^a^
Yes	12 (37.5%)	7 (26.9%)	19 (32.7%)	
No	20 (62.5%)	19 (73.1%)	39 (67.3%)	
**IOP > 21 mmHg or Antiglaucoma Medication before SOR**				1.000^a^
Yes	9 (15.5%)	7 (12.0%)	16 (27.5%)	
No	23 (84.5%)	19 (88.0%)	42 (72.5%)	

ROSO: removal of silicone oil; BCVA: best corrected visual acuity; CME: cystoid macular edema; ERM: epiretinal membrane; IOP: intraocular pressure

ROSO surgery sometimes included additional procedures such as laser photocoagulation, ERM peeling, and peripheral membrane peeling, with comparable frequencies between groups. Air-fluid exchange without gas endotamponade was performed in the majority of cases (47 out of 58 eyes), while C3F8 gas, and SF6 gas, were used in 9 and 2 cases, respectively (Table 4).

**Table 4:** Characteristics of ROSO surgery

	Group I [min, 3 Months] *N* (= 32) *N* (%)	Group II [3 Months, max] *N* (= 26) *N* (%)	Total (*N* = 58) *N* (%)	*P* value
**Type of Endotamponade after ROSO**				0.989^c^
Air	26 (81.2%)	21 (80.8%)	47 (81.0%)	
C3F8	5 (15.6%)	4 (15.4%)	9 (15.5%)	
SF6	1 (3.1%)	1 (3.8%)	2 (3.4%)	
**Laser Photocoagulation at ROSO**				0.550^a^
Performed	7 (21.8%)	8 (30.7%)	15 (25.9%)	
Not Performed	25 (78,2%)	18 (69.3%)	43 (74.1%)	
**ERM Peeling**				1.000^a^
Performed	11 (34.3%)	3 (11.5%)	14 (15%)	
Not Performed	21 (65.7%)	23 (88.5%)	44 (75.8%)	
**Peeling of Peripheral Membranes at ROSO**				0.740^a^
Performed	5 (15.7%)	5 (19.2%)	10 (17.2%)	
Not Performed	27 (84.3%)	21 (80.7%)	48 (82.8%)	
**Surgeon**				1.000^a^
C.S.	32 (100.0%)	26 (100.0%)	58 (100.0%)	
M.S.	0 (0.0%)	0 (0.0%)	0 (0.0%)	

RD: retinal detachment; ROSO: removal of silicone oil; ERM: epiretinal membrane; a: Fisher’s exact test; c: chi-square test

### Clinical findings after ROSO

#### Retinal redetachment

**Fig. 2:**
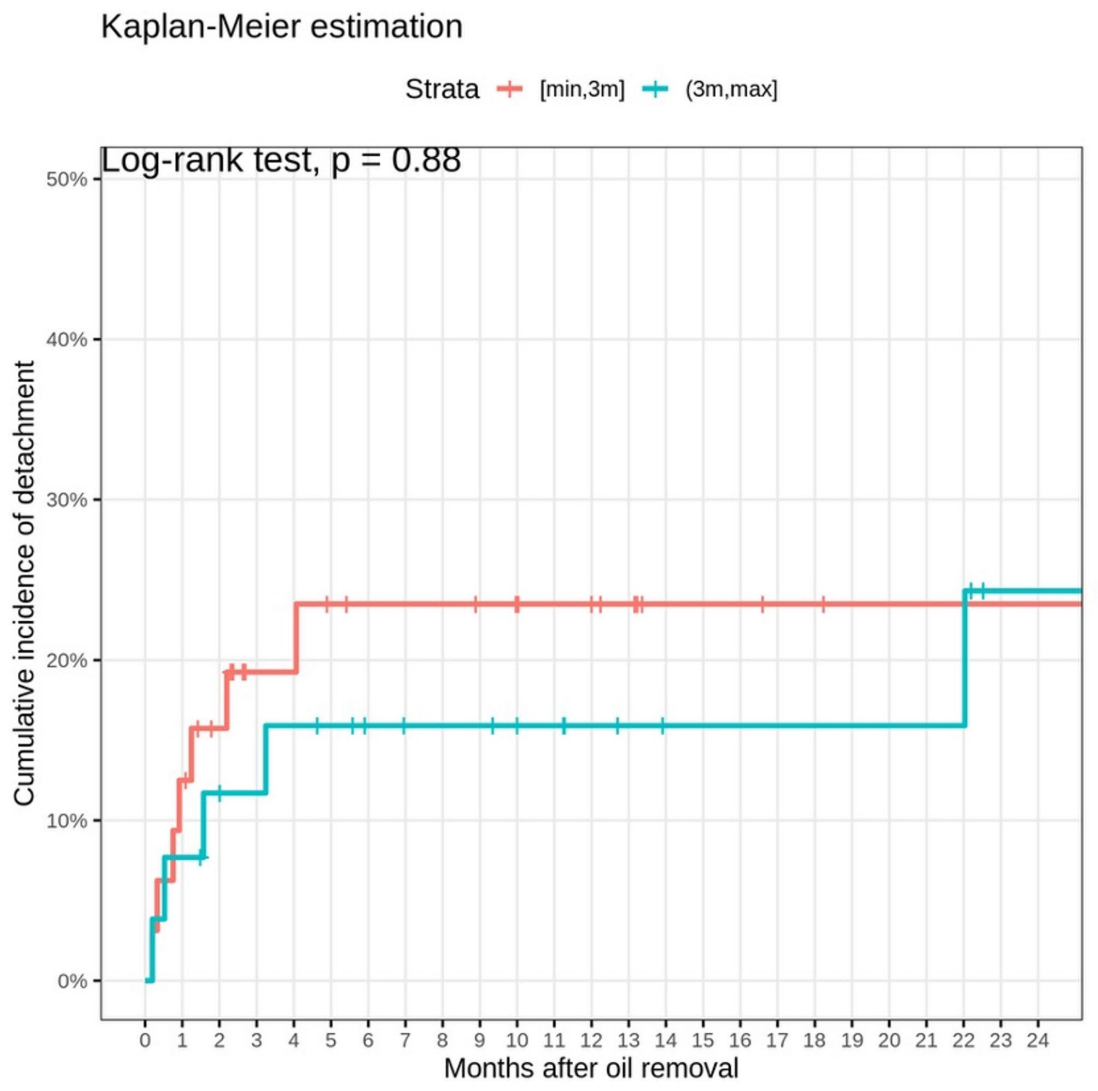
Kaplan-Meier estimation of the cumulative incidence of retinal redetachment in Group I (red line) and Group II (blue line) in the months following silicone oil removal. Most redetachments occur in the first 20 months after silicone oil removal. No statistically significant difference in the incidence of redetachment is note between the two groups (Log-rank Test: *p* = 0.88)

Retinal redetachment was observed in 13 cases overall. The rates of retinal redetachment were comparable between the two groups (Group I: *n* = 7 (21.9%, 95% CI [9.3%-40%]); Group II: *n* = 6 (23.1%, 95% CI [8.9%-43.6%]), *p* = 1.00). A longer SOE did not appear to protect against macular involvement upon the first recurrent RD (Group I: *n* = 4 macula-off; in Group II: *n* = 1 macula-off, *p* = 0.26), or against the number of subsequent redetachments (Group I mean: 0.38±0.87); Group II mean: 0.27±0.53 (*p* = 0.98). Kaplan-Meier analysis (Fig. 2) showed no significant difference in the cumulative incidence of Re-RD (Log-rank Test: *p* = 0.88) between groups. Cumulative incidence of Re-RDs at 24 months given by Group I: *n* = 7 (23.6%, 95% CI [6.4%-37.5%]) and Group II: *n* = 6 (24.3%, 95% CI [1.08%-42.1%]). No significant correlation was found between the time to ROSO and the number of redetachments (Spearman´s ρ = − 0.20, *p* = 0.12).

#### Functional changes, CME, ERM and IOP

Visual acuity development and complications following ROSO are summarized in Table 5. In Group I, BCVA improved from 1.1 ± 0.8 logMar (range: 0.10–2.40) at presentation to 0.8 ± 0.5 (range: 0.1–2.4) logMar at last follow-up. In Group II, BCVA improved from 1.7 ± 0.8 (range: 0.2–2.7) logMAR at presentation to 0.8 ± 0.7 (range: 0.1- 3.0) logMAR at last follow-up. Macula-off was observed in 22 eyes in Group I and 17 eyes in Group II at the time of the primary retinal detachment (*p* = 1.00). The Wilcoxon Rank Sum Test indicated comparable BCVA outcomes between the two groups after ROSO (*p* = 0.36). Postoperative incidences of CME (Group I: *n* = 13 (40.6%, 95% CI [25.5%–57.7%]), Group II: *n* = 9 (34.6%, 95% CI [19.4%–53.8%]), *p* = 0.78), ERM (Group I: *n* = 13 (40.6%, 95% CI [25.5%–57.7%]) Group II: *n* = 7 (26.9%, 95% CI [13.7%–46.1%], *p* = 0.40), and elevated intraocular pressure (IOP) (Group I: *n* = 7 (21.9%, 95% CI [11.0%–38.8%]), Group II: *n* = 7 (26.9%, 95% CI [13.7%–46.1%], *p* = 0.76) were also similar between groups.

**Table 5:** Characteristics after ROSO surgery

	Group I [min, 3 Months] *N* (= 32) *N* (%)	Group II [3 Months, max] *N* (= 26) *N* (%)	Total (*N* = 58) *N* (%)	*P* value
**Recurrent RD after ROSO**				1.000^a^
Yes	7 (21.9%)	6 (23.1%)	13 (22.4%)	
No	25 (78.1%)	20 (76.9%)	45 (77.6%)	
**Macula Involvement** **(First Re-RD after ROSO)**				0.266^a^
Macula- Off	4 (57.1%)	1 (16.7%)	5 (38.5%)	
Macula-On	3 (42.9%)	5 (83.3%)	8 (61.5%)	
**Number of recurrent RDs after ROSO**				0.983^b^
Range	0.00–4.00	0.00–2.00	0.00–4.00	
Mean (SD)	0.38 (0.87)	0.27 (0.53)	0.33 (0.73)	
Median (Q1, Q3)	0.00 (0.00, 0.00)	0.00 (0.00, 1.00)	0.00 (0.00, 0.00)	
**IOP > 21 mmHg or IOP-lowering medications after ROSO**				0.761^a^
Yes	7 (21.9%)	7 (26.9%)	14 (24.1%)	
No	25 (78.1%)	19 (73.1%)	44 (75.9%)	
**CME after ROSO**				0.787^a^
Yes	13 (40.6%)	9 (34.6%)	22 (37.9%)	
No	19 (59.4%)	17 (65.4%)	36 (62.1%)	
**ERM after ROSO**				0.405^a^
Yes	13 (40.7%)	7 (26.9%)	20 (34.4%)	
No	19 (59.3%)	19 (73.1%)	38 (65.6%)	
**BCVA at last Follow-up** **(LogMar)**				0.366^b^
Range	0.10–2.40	0.10–3.00	0.10–3.00	
Mean (SD)	0.89 (0.55)	0.86 (0.72)	0.88 (0.63)	
Median (Q1, Q3)	0.75 (0.47, 1.124)	0.55 (0.40, 1.17)	0.70 (0.40, 1.22)	

ROSO: removal of silicone oil; RD: retinal detachment; IOP: intraocular pressure; CME: cystoid macular edema; ERM: epiretinal membrane; BCVA: best corrected visual acuity; a: Fisher’s exact test; b: Mann-Whitney test

#### Group matching

For matching, we included 52 from the 58 eyes of the original dataset in order to include only one eye per patient. Among the 5 patients who contributed both eyes, only one eye was randomly selected per patient, resulting in the exclusion of 5 eyes. In addition, one eye was excluded due to missing information on macular status at the time of index repair. Thus, for matching we considered 52 eyes. Different matching methods were tested, and the cardinality method achieved the best balance and the largest sample size. This yielded 11 well-balanced pairs (22 eyes), with all standardized mean differences below 0.1, indicating excellent covariate balance between groups. Consistent with the findings from the unmatched analysis, there were no differences in baseline characteristics between the two groups, and the incidence of the primary outcome, retinal redetachment, did not differ significantly between the matched groups (Group I: *n* = 2 (18% 95%-CI [2.3%-52%]); Group II: *n* = 3 (27.3% 95%-CI [6.0%-61.0%]), *p* = 1.00 (Supplemental Table 1). It should be noted that very low sample size extremely underpowers any statistical comparison. For example, for a difference of 25% between the groups, assuming the level of significance of 0.05 and power of 80% for Fisher’s Exact Test we would need between 33 and 57 (depending on the true incidences in Groups I and II) eyes per group.

## Discussion

The use of silicone oil endotamponade represents an effective treatment strategy in the management of complicated retinal detachment, achieving anatomic success rates ranging from 72 to 100% [13, 21–24]. In the published literature, recurrence of retinal detachment after silicone oil has been reported at incidences between 5 and 31.4% [14]. Although the duration of silicone oil endotamponade does not appear to influence the final anatomical success [13, 24, 25], some studies have reported a slightly increased retinal detachment rate with an endotamponade duration of less than three months [8, 9, 16]. This difference in retinal redetachment incidence has been attributed to PVR, which has a median onset of two months following primary ppV surgery [26]. In line with existing literature, the cumulative incidence of redetachment in our cohort was 22.4%, with comparable rates between patients who underwent ROSO before 3 months and those who had ROSO after 3 months. This aligns with studies supporting both anatomical and functional success even with shorter SOE durations [12–14].

The three-months cut-off was selected based on thresholds commonly used in previous studies and the widely practiced approach that considers 3 months as the standard duration for SOE [8, 11]. However, significant variability exists among studies, with some reporting cut-offs as short as two months or as long as six months [13, 27].

Silicone oil emulsification appears to be one of the most important risk factors for SO-related complications. Although reports indicate that SO emulsification can occur between 4.3 and 5 months postoperatively [28, 29], the first signs have been reported as early as 2 months after silicone oil implantation [15]. Increased intraocular pressure, SORVL as well as influence in the central macular thickness have been associated with increased SOE duration, rendering an earlier removal an attractive option in certain cases [6, 24].

In the present study, increased IOP was reported in 16 cases (27.5%) following SO injection, consistent with the reported incidence of 3–40% in previous studies [30, 31]. Elevated IOP persisted after SO removal in 14 cases but was effectively managed with topical IOP-lowering medications, without the need for glaucoma surgery or secondary surgical interventions. Although some studies suggest that the duration of SO endotamponade does not affect ocular hypertension [11, 32], other evidence links prolonged tamponade and elevated intraocular pressure to unexplained visual loss [6]. One proposed mechanism is that longer SO retention increases the likelihood of emulsification, which may in turn contribute to IOP elevation, though this remains speculative [6, 32].

Several case series have reported the incidence of unexplained visual loss after SO injection [6, 33–38]. In the study of Scheerlinck et al., unexplained visual loss was reported in almost 30% of patients treated with SO injection following macula-on retinal detachment. Duration of SOE was the only statistically significant factor associated with visual loss [6]. Thinning of the inner retinal layers has also been described in SO eyes with SORVL [34, 39, 40]. A recent meta-analysis evaluating the impact of silicone oil tamponade on the retinal layer and choroidal thickness identified a significant reduction in central macular thickness after SO removal, correlated with the duration of SO tamponade [41]. In our study, no statistically significant differences were observed in the incidence of increased IOP and visual acuity between early and late ROSO groups at the end of follow-up. Group I demonstrated slightly better visual acuity at baseline and prior to ROSO. Although this difference was not statistically significant, it may suggest that Group I included relatively less complicated cases, in which earlier oil removal was considered safer. The final VA at the last follow-up was comparable between the two groups, and both showed an improvement in mean BCVA after SO removal compared to baseline presentation.

Additionally, there were no statistically significant differences in the rates of ERM formation or CME related to the duration of endotamponade. However, a study of 65 eyes by Er et al. reported an increased incidence of macular edema in eyes with SOE durations exceeding three months [42].

While our findings provide important early insights, they should be interpreted with caution. Given the retrospective observational design, relatively small sample size impeding multivariate modeling, and potential for residual confounding, the results suggest that no clear differences were observed between groups rather than establishing non-inferiority. Important sources of bias include confounding by indication, sample size constraints, and the limited external validity inherent in a single-center study. Nonetheless, our statistical and cardinality matching analysis carefully accounted for several key surgical and clinical factors, including silicone oil viscosity, adjunctive procedures, and case selection, thereby strengthening internal validity. Moreover, this study represents the first clinical evidence stratified by timing of oil extraction, offering a valuable foundation for future larger, prospective studies. As such, our results provide an important first step toward generating more robust predictive models that can better inform clinical decision-making in broader populations.

## Conclusion

In conclusion, our results support the consideration of early ROSO (≤ 3 months) as a feasible and safe option in appropriately selected patients, without compromising retinal reattachment rates, visual outcomes, or the incidence of postoperative complications. Further prospective studies with larger cohorts are warranted to validate these findings and to refine guidelines regarding the optimal timing of silicone oil removal in retinal detachment surgery.

## Supplementary Information

Below is the link to the electronic supplementary material.

Supplementary Material 1

## Data Availability

The datasets generated and analyzed during the current study are not publicly available due to patient confidentiality and ethical restrictions but are available from the corresponding author upon reasonable request. Access to the data is subject to approval by the Institutional Review Board of the Hamburg Medical Chamber (Ethik-Kommission der Ärztekammer Hamburg).

## Abbreviations

SO: Silicone Oil
RD: Retinal Detachment
RRD: Rhegmatogenous Retinal Detachment
SF₆: Sulfur Hexafluoride
C₃F₈: Perfluoropropane
SOE: Silicone Oil Endotamponade
PVR: Proliferative Vitreoretinopathy
ROSO: Removal of Silicone Oil
SORVL: Silicone Oil-Related Visual Loss
IOP: Intraocular Pressure
ppV: Pars Plana Vitrectomy
BCVA: Best-Corrected Visual Acuity
OCT: Optical Coherence Tomography
CME: Cystoid Macular Edema
ERM: Epiretinal Membrane
ANOVA: Analysis of Variance
Re-RD: Recurrent Retinal Detachment
