# Supplementary material for: Early versus late silicone oil tamponade removal after rhegmatogenous retinal detachment: a retrospective real world comparative study
Source: Int J Retina Vitreous. 2025 Oct 13;11:105. doi: 10.1186/s40942-025-00743-9 (PMC12516905; doi:10.1186/s40942-025-00743-9)
Supplement: Supplementary file 1 — Supplementary Material 1 [file 40942_2025_743_MOESM1_ESM.docx]

| **Supplemental Table 1: Preoperative and postoperative characteristics of Group I and II after group matching** | | | | | |
| --- | --- | --- | --- | --- | --- |
|  | **Matched Group I**  **[min, 3 Months]**  **(N=11)**  **N (%)** | **Matched Group II**  **(3 Months, max]**  **(N=11)**  **N (%)** | **Total**  **(N=22)**  **N (%)** | **P value** |  |
| **Sex (Eyes)** |  |  |  | 1.000^a^ |  |
| Female | 5 (45.5%) | 4 (36.4%) | 9 (40.9%) |  |  |
| Male | 6 (54.5%) | 7 (63.6%) | 13 (59.1%) |  |  |
| **Age (years)** |  |  |  | 0.341^b^ |  |
| Range | 38.46 - 85.49 | 52.44 - 87.03 | 38.46 - 87.03 |  |  |
| Mean (SD) | 68.07 (15.57) | 66.28 (10.06) | 67.18 (12.82) |  |  |
| Median (Q1, Q3) | 71.60 (63.05, 78.96) | 63.49 (60.44, 70.62) | 68.86 (60.49, 78.22) |  |  |
| **Macula Involvement**  **(Primary Retinal Detachment)** |  |  |  | 1.000^a^ |  |
| Macula-Off | 7 (63.6%) | 7 (63.6%) | 14 (63.6%) |  |  |
| Macula-On | 4 (36.4%) | 4 (36.4%) | 8 (36.4%) |  |  |
| **Months to SOR** |  |  |  | 1.000^a^ |  |
| Range | 1.00 - 3.00 | 4.00 - 5.00 | 1.00 - 5.00 |  |  |
| Mean (SD) | 2.45 (0.69) | 4.09 (0.30) | 3.27 (0.98) |  |  |
| Median (Q1, Q3) | 3.00 (2.00, 3.00) | 4.00 (4.00, 4.00) | 3.50 (3.00, 4.00) |  |  |
| **Bullous RRD** |  |  |  | 1.000^a^ |  |
| Yes | 5 (45.5%) | 5 (45.5%) | 10 (45.5%) |  |  |
| No | 6 (54.5%) | 6 (54.5%) | 12 (54.5%) |  |  |
| **PVR Grade** |  |  |  | 1.000^a^ |  |
| A | 8 (72.7%) | 8 (72.7%) | 16 (72.7%) |  |  |
| B | 1 (9.1%) | 1 (9.1%) | 2 (9.1%) |  |  |
| C | 2 (18.2%) | 2 (18.2%) | 4 (18.2%) |  |  |
| **Retinal Quadrant Involved (n)** |  |  |  | 0.780^b^ |  |
| Range | 1.00 - 4.00 | 1.00 - 4.00 | 1.00 - 4.00 |  |  |
| Mean (SD) | 2.00 (0.77) | 2.00 (0.63) | 2.00 (0.69) |  |  |
| Median (Q1, Q3) | 2.00 (2.00, 2.00) | 2.00 (2.00, 2.00) | 2.00 (2.00, 2.00) |  |  |
| **Retinal Breaks (n)** |  |  |  | 0.944^b^ |  |
| Range | 0.00 - 5.00 | 0.00 - 5.00 | 0.00 - 5.00 |  |  |
| Mean (SD) | 1.18 (1.54) | 1.18 (1.60) | 1.18 (1.53) |  |  |
| Median (Q1, Q3) | 1.00 (0.00, 2.00) | 1.00 (0.00, 1.50) | 1.00 (0.00, 2.00) |  |  |
| **SO Viscosity** |  |  |  | 1.000^a^ |  |
| 2000 | 8 (72.7%) | 8 (72.7%) | 16 (72.7%) |  |  |
| 5000 | 3 (27.3%) | 3 (27.3%) | 6 (27.3%) |  |  |
| **Type of Endotamponade after SOR** |  |  |  | 1.000^a^ |  |
| Air | 10 (90.9%) | 10 (90.9%) | 20 (90.9%) |  |  |
| C3F8 | 1 (9.1%) | 1 (9.1%) | 2 (9.1%) |  |  |
| **Recurrent RD after SOR** |  |  |  | 1.000^a^ |  |
| Yes | 2 (18.2%) | 3 (27.3%) | 5 (22.7%) |  |  |
| No | 9 (81.8%) | 8 (72.7%) | 17 (77.3%) |  |  |
| **Number of recurrent RDs after SOR** |  |  |  | 0.654^b^ |  |
| Range | 0.00 - 2.00 | 0.00 - 2.00 | 0.00 - 2.00 |  |  |
| Mean (SD) | 0.27 (0.65) | 0.36 (0.67) | 0.32 (0.65) |  |  |
| Median (Q1, Q3) | 0.00 (0.00, 0.00) | 0.00 (0.00, 0.50) | 0.00 (0.00, 0.00) |  |  |
| **Macula Involvement**  **(Recurrent RD)** |  |  |  | 0.400^a^ |  |
| Yes | 0 (0.0%) | 2 (66.7%) | 2 (40.0%) |  |  |
| No | 2 (100.0%) | 1 (33.3%) | 3 (60.0%) |  |  |
| **CME before SOR** |  |  |  | 0.635^a^ |  |
| Yes | 4 (36.4%) | 2 (18.2%) | 6 (27.3%) |  |  |
| No | 7 (63.6%) | 9 (81.8%) | 16 (72.7%) |  |  |
| **CME after SOR** |  |  |  | 1.000^a^ |  |
| Yes | 4 (36.4%) | 3 (27.3%) | 7 (31.8%) |  |  |
| No | 7 (63.6%) | 8 (72.7%) | 15 (68.2%) |  |  |
| **BCVA at presentation**  **(LogMar)** |  |  |  | 0.741^b^ |  |
| Range | 0.10 - 2.40 | 0.30 - 2.30 | 0.10 - 2.40 |  |  |
| Mean (SD) | 1.31 (0.94) | 1.28 (0.79) | 1.30 (0.85) |  |  |
| Median (Q1, Q3) | 1.50 (0.55, 2.30) | 1.00 (0.60, 2.10) | 1.20 (0.60, 2.25) |  |  |
| **BCVA before SOR**  **(LogMar)** |  |  |  | 0.467^b^ |  |
| Range | 0.20 - 1.30 | 0.00 - 1.30 | 0.00 - 1.30 |  |  |
| Mean (SD) | 0.61 (0.30) | 0.71 (0.39) | 0.66 (0.34) |  |  |
| Median (Q1, Q3) | 0.60 (0.50, 0.75) | 0.70 (0.45, 1.00) | 0.60 (0.50, 0.95) |  |  |
| **BCVA at last Follow-up**  **(LogMar)** |  |  |  | 0.371^b^ |  |
| Range | 0.10 - 2.40 | 0.10 - 2.40 | 0.10 - 2.40 |  |  |
| Mean (SD) | 0.87 (0.65) | 0.70 (0.65) | 0.79 (0.64) |  |  |
| Median (Q1, Q3) | 0.70 (0.40, 1.10) | 0.40 (0.35, 0.81) | 0.65 (0.40, 1.00) |  |  |
| **IOP> 21 mmHg or IOP-lowering medications before SOR** |  |  |  | 1.000^a^ |  |
| Yes | 3 (27.3%) | 2 (18.2%) | 5 (22.7%) |  |  |
| No | 8 (72.7%) | 9 (81.8%) | 17 (77.3%) |  |  |
| **IOP> 21 mmHg or IOP-lowering medications after SOR** |  |  |  | 1.000^a^ |  |
| Yes | 2 (18.2%) | 2 (18.2%) | 4 (18.2%) |  |  |
| No | 9 (81.8%) | 9 (81.8%) | 18 (81.8%) |  |  |
| **Surgeon who performed primary surgery** |  |  |  | 1.000^a^ |  |
| C.S. | 11 (100.0%) | 11 (100.0%) | 22 (100.0%) |  |  |
| M.S. | 0 (0.0%) | 0 (0.0%) | 0 (0.0%) |  |  |
| **Surgeon who performed SOR** |  |  |  | 1.000^a^ |  |
| C.S. | 11 (100.0%) | 11 (100.0%) | 22 (100.0%) |  |  |
| M.S. | 0 (0.0%) | 0 (0.0%) | 0 (0.0%) |  |  |
| **Pefluorocarbon liquid (PCFL) at primary surgery** |  |  |  | 0.586^a^ |  |
| Yes | 2 (20.0%) | 1 (9.1%) | 3 (14.3%) |  |  |
| No | 8 (80.0%) | 10 (90.9%) | 18 (85.7%) |  |  |
| **Presence of vitreous hemorrhage at primary surgery** |  |  |  | 1.000^a^ |  |
| Yes | 0 (0.0%) | 0 (0.0%) | 0 (0.0%) |  |  |
| No | 11 (100.0%) | 9 (100.0%) | 20 (100.0%) |  |  |
| **Retinotomy at primary surgery** |  |  |  | 1.000^a^ |  |
| Yes | 0 (0.0%) | 0 (0.0%) | 0 (0.0%) |  |  |
| No | 11 (100.0%) | 11 (100.0%) | 22 (100.0%) |  |  |
| **Retinectomy at primary surgery** |  |  |  | 1.000^a^ |  |
| Yes | 0 (0.0%) | 0 (0.0%) | 0 (0.0%) |  |  |
| No | 11 (100.0%) | 11 (100.0%) | 22 (100.0%) |  |  |
| **ERM Peeling at SOR** |  |  |  | 1.000^a^ |  |
| Yes | 2 (18.2%) | 2 (18.2%) | 4 (18.2%) |  |  |
| No | 9 (81.8%) | 9 (81.8%) | 18 (81.8%) |  |  |
| **Peeling of Peripheral Membranes at SOR** |  |  |  | 1.000^a^ |  |
| Yes | 1 (9.1%) | 1 (9.1%) | 2 (9.1%) |  |  |
| No | 10 (90.9%) | 10 (90.9%) | 20 (90.9%) |  |  |
| **Follow-up (months)** |  |  |  | 0.261^b^ |  |
| Range | 3.0 - 47.0 | 3.0 - 46.0 | 3.0 - 47.0 |  |  |
| Mean (SD) | 21.0 (14.6) | 14.8 (13.6) | 17.9 (14.1) |  |  |
| Median (Q1, Q3) | 18.0 (9.5, 31.5) | 10.0 (4.5, 23.0) | 15.0 (6.0, 26.2) |  |  |
| BCVA: best corrected visual acuity; RD: retinal detachment; PVR: proliferative vitreoretinopathy; RD: retinal detachment; SOR: silicone oil removal; CME: cystoid macular edema; ERM: epiretinal membrane; IOP: intraocular pressure. a: Fisher's exact test; b: Mann-Whitney test. | | | | | |
